# Natural Products for Rheumatoid Arthritis Therapy: Research Progress and Translational Perspectives

**DOI:** 10.3390/ijms27219835

**Published:** 2026-11-02

**Authors:** Qinxuan Zhou, Zhengyang Song, Bing Xu, Canyang Zhang, Xin-Hui Xing, Yi Wang

**Affiliations:** 1 Institute of Biopharmaceutical and Health Engineering, Shenzhen International Graduate School, Tsinghua University, Shenzhen 518055, China; zhouqx24@mails.tsinghua.edu.cn (Q.Z.); songzhen23@mails.tsinghua.edu.cn (Z.S.); bingxu@sz.tsinghua.edu.cn (B.X.); zhang.cy@sz.tsinghua.edu.cn (C.Z.); 2 Key Laboratory of Active Proteins and Peptides Green Biomanufacturing of Guangdong Higher Education Institutes, Shenzhen International Graduate School, Tsinghua University, Shenzhen 518055, China; 3 Institute of Biomedical Health Technology and Engineering, Shenzhen Bay Laboratory, Shenzhen 518118, China; 4 Key Laboratory for Industrial Biocatalysis (Ministry of Education), Institute of Biochemical Engineering, Department of Chemical Engineering, Tsinghua University, Beijing 100084, China; 5 Center for Synthetic and Systems Biology, Tsinghua University, Beijing 100084, China

**Keywords:** rheumatoid arthritis, natural products, extraction techniques, isolation and purification, biological origin, anti-inflammatory mechanisms, immune modulation, drug delivery systems

## Abstract

Rheumatoid arthritis (RA) is a chronic autoimmune disease characterized by persistent synovial inflammation, progressive joint destruction, and substantial systemic complications. Although current pharmacological therapies have improved disease control, important unmet needs for RA remission remain owing to the incomplete response, loss of efficacy, long-term safety concerns, limited capacity for tissue repair, and barriers related to accessibility and adherence. Against this background, natural products have emerged as an important area of interest in RA therapy research because many of them can modulate multiple components of the pathogenic network, including immune dysregulation, synovial inflammation, oxidative stress, and structural damage. Therefore, this review provides an updated overview of natural products for RA treatment, with emphasis on their therapeutic effects, mechanistic basis, and translational potential as well as key challenges. In particular, we discuss the natural-product research in RA therapy along an evolving continuum, from traditionally used complex natural products, such as herbal medicines and venom-based preparations, to structurally defined bioactive compounds, including flavonoids, alkaloids, terpenoids, and polysaccharides. The review further summarizes the principal pharmacological actions of these agents, their advantages and limitations in anti-RA intervention, and current strategies to enhance their development, including formulation optimization, delivery systems, and mechanism-guided translational approaches.

## 1. Introduction

Rheumatoid arthritis (RA) is a chronic systemic autoimmune disease that primarily affects synovial joints, leading to persistent inflammation, progressive cartilage and bone destruction, and ultimately joint deformity, functional decline, and disability [1]. Affecting approximately 0.25–1% of the global population, RA disproportionately burdens women and individuals in their most productive years of life [2]. Beyond articular damage, RA is frequently accompanied by extra-articular complications and comorbidities, particularly involving the cardiovascular, respiratory, and nervous systems, which contribute substantially to excess mortality and overall disease burden [2]. With its rising global prevalence [3] and profound impact on quality of life, disability, and healthcare systems, RA remains a major therapeutic challenge and a persistent priority in drug discovery and translational research.

Despite substantial advances in RA management, the disease remains an incurable chronic autoimmune disorder, and durable disease control is still not achieved in a considerable proportion of patients [3]. Current therapeutic approaches have significantly improved clinical outcomes, yet they continue to be limited by incomplete efficacy, safety concerns, and challenges associated with long-term use and accessibility. These persistent unmet needs underscore the necessity for new therapeutic strategies that can more effectively address the complex pathogenic network of RA while offering improved efficacy, safety and translational potential. In this context, natural products have emerged as an important area of interest owing to their structural diversity, multitarget activities, and potential applicability in long-term RA intervention.

Natural products derived from plants, animals, and microbes, including fungi, have long played a vital role in the treatment of inflammatory and autoimmune diseases, showing particular promise for RA management. These compounds exhibit remarkable structural diversity and multifaceted mechanisms of action. They are often capable of modulating multiple signaling pathways simultaneously—such as NF-κB, JAK/STAT, MAPK, and PI3K/AKT—while also regulating various immune cell subsets, including T helper 17 (Th17) cells, regulatory T (Treg) cells, and macrophages [4]. This multi-target pharmacological profile aligns well with the complex, multifactorial pathogenesis of RA and distinguishes natural products from conventional single-target synthetic drugs, offering the potential for broader disease control with fewer off-target effects, although the limited clinical benefit of IL-17 blockade underscores that modulation of disease-associated pathways does not necessarily translate into therapeutic efficacy [5]. Indeed, a variety of natural compounds have demonstrated significant anti-RA activity in both preclinical and clinical studies. These include triptolide and celastrol from *Tripterygium wilfordii*, melittin from bee venom, polysaccharides from the medicinal fungus Ganoderma lucidum, and omega-3 fatty acids from marine sources [6]. With the integration of modern pharmacology, nanotechnology, and systems biology, natural products are increasingly transitioning from traditional adjunct therapies to novel therapeutic agents and synergistic interventions. Their applications are expanding, ranging from monotherapy to combination with conventional DMARDs (disease-modifying antirheumatic drugs), advanced drug delivery systems, or use as nutraceuticals—reflecting the continuous evolution of RA treatment paradigms toward greater diversity and precision.

In this context, this review provides a narrative overview of natural products for the treatment of RA. We first summarize the pathogenesis of RA and the limitations of current therapeutic strategies, and then examine natural products along an evolving research continuum, from traditionally used complex natural products to structurally defined bioactive compounds. We further discuss their therapeutic effects, mechanistic basis, and potential applications in advanced delivery systems and combination regimens, with particular emphasis on translational prospects and the key challenges for clinical development.

## 2. Pathogenesis and Therapeutic Strategies of Rheumatoid Arthritis

### 2.1. Pathogenesis of Rheumatoid Arthritis

RA is a multifactorial autoimmune disease arising from the interplay between genetic susceptibility and environmental triggers. HLA-DRB1 shared epitope alleles represent the strongest genetic risk factor, particularly in ACPA (anti-citrullinated protein autoantibodies)-positive RA [7], while other loci, such as PTPN22 [8] and CTLA4 [9], further implicate dysregulated adaptive immunity in disease initiation. Environmental exposures, including smoking, silica exposure, mucosal infection, periodontal pathogens, and gut microbial dysbiosis, may promote aberrant citrullination and loss of immune tolerance in genetically susceptible individuals [10]. Together, these gene–environment interactions drive the generation of ACPA and rheumatoid factor, thereby establishing the immunologic basis for disease initiation and shaping the clinical and molecular heterogeneity of RA.

Following the loss of immune tolerance, RA evolves into a state of persistent synovial inflammation sustained by reciprocal crosstalk among immune cells, stromal cells, and osteoclastogenic signals [11]. Leukocyte recruitment into the synovium is driven by endothelial activation, chemokine gradients, and pathological angiogenesis, creating a remodeled microenvironment in which innate and adaptive immune responses act in concert. Within this niche, antigen-presenting cells activate autoreactive T cells, B cells amplify local immunity, while myeloid cells, particularly macrophages, sustain the inflammatory cascades [12]. An imbalance between pro-inflammatory Th17 cells and functionally impaired regulatory T cells further skews the synovial milieu toward chronic inflammation [13,14,15]. Central cytokine networks, especially those driven by TNF-α, IL-1, IL-6, and IL-17, together with intracellular pathways such as JAK/STAT and NF-κB [16,17], reinforce inflammatory persistence and stromal activation. In parallel, fibroblast-like synoviocytes acquire an aggressive, apoptosis-resistant phenotype [18] that drives synovial hyperplasia, sustains inflammatory cell persistence, and directly contributes to tissue injury, thereby establishing a self-perpetuating inflammatory niche [19].

Persistent synovitis ultimately culminates in progressive structural damage involving both cartilage and bone. Hyperplastic synovium and activated fibroblast-like synoviocytes promote cartilage destruction through invasive behavior and the release of matrix-degrading enzymes, including MMPs (matrix metalloproteinases) and ADAMTS (a disintegrin and metalloproteinase with thrombospondin motifs), while inflammatory mediators such as IL-1 and IL-17A impair chondrocyte survival and matrix homeostasis [18]. In parallel, bone erosion is driven primarily by osteoclastogenesis, promoted by RANKL (receptor activator of NF-κB ligand) and macrophage colony-stimulating factor, and further amplified by pro-inflammatory cytokines. At the same time, chronic inflammation markedly restricts periarticular bone repair by suppressing osteogenic differentiation, thereby favoring irreversible joint destruction [20]. In addition to local joint destruction, chronic inflammation in RA exerts systemic effects, contributing to cardiovascular complications, metabolic dysregulation, sarcopenia, pulmonary involvement, and osteoporosis [21].

**Figure 1:**
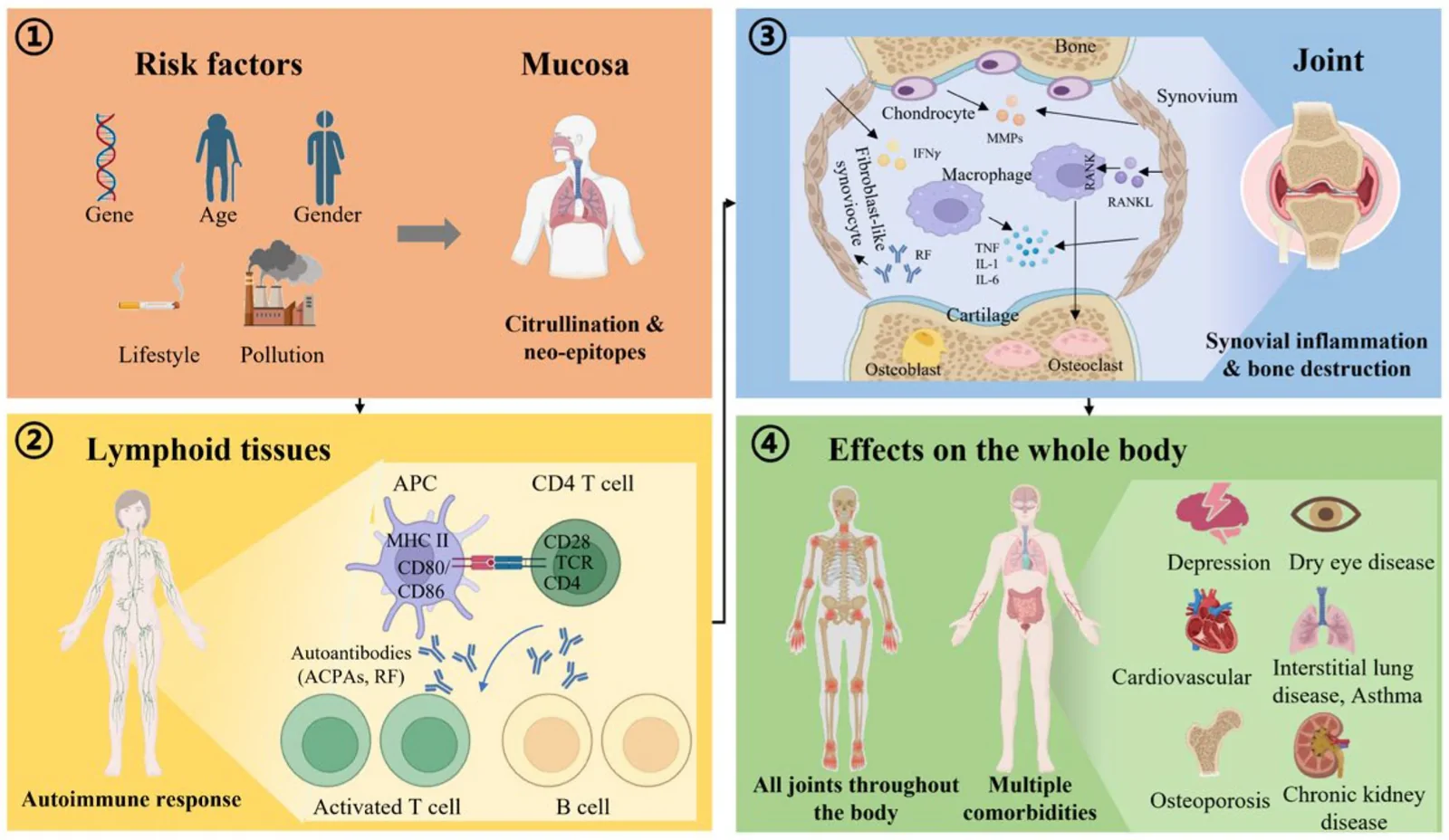
Pathogenesis of RA. ➀ Genetic susceptibility, age, sex, and environmental factors contribute to disease susceptibility. Mucosal processes, including protein citrullination and neoepitope formation, contribute to the initiation of autoimmunity. ➁ In lymphoid tissues, antigen presentation and costimulatory signaling promote T-cell activation and B-cell responses, including the production of autoantibodies such as ACPAs and RF. ➂ Within the joint, interactions among immune cells, fibroblast-like synoviocytes, and chondrocytes sustain synovial inflammation. Inflammatory cytokines and MMPs contribute to cartilage degradation, while RANKL-mediated osteoclast activation promotes bone erosion. ➃ Disease manifestations extend beyond individual joints to polyarticular involvement and systemic comorbidities, including cardiovascular and pulmonary disease, osteoporosis, chronic kidney disease, dry eye disease, and depression. Arrows in panels ➁ and ➂ indicate the direction of cellular interactions and downstream biological effects.

Collectively, RA pathogenesis is orchestrated by a highly interconnected immune–stromal network characterized by molecular heterogeneity, signaling redundancy, and progressive tissue-destructive responses (Figure 1). These features not only underlie the chronicity and complexity of RA, but also help explain why current therapeutic strategies, despite considerable advances, often fail to achieve sustained remission, restore immune homeostasis, or prevent progressive tissue damage in a substantial proportion of patients.

### 2.2. Limitations of Current Therapeutic Strategies for Rheumatoid Arthritis

Current RA management is guided by the principles of early intervention and treat-to-target, with the goal of achieving sustained remission or, at minimum, low disease activity [22]. In clinical practice, the treatment relies on the stepwise and individualized use of nonsteroidal anti-inflammatory drugs (NSAIDs), glucocorticoids, conventional synthetic disease-modifying antirheumatic drugs (csDMARDs), biologic DMARDs (bDMARDs), and targeted synthetic DMARDs (tsDMARDs) [23,24]. NSAIDs and glucocorticoids are mainly used for rapid symptom relief and short-term inflammatory control, whereas csDMARDs—particularly methotrexate, which remains the anchor drug—constitute the backbone of long-term disease-modifying therapy [25]. For patients with an inadequate response to csDMARDs, escalation to bDMARDs targeting cytokines or immune cell pathways, or to tsDMARDs such as Janus kinase (JAK) inhibitors [26,27], has substantially expanded therapeutic options. These strategies have improved symptom control, suppressed inflammatory activity, and slowed structural progression in many patients. Nevertheless, important unmet needs remain in terms of efficacy, durable immune control, tissue repair, long-term safety, and real-world treatment feasibility.

Despite advances in RA treatment, more than 40% of patients in a Finnish early-RA cohort did not achieve sustained remission, defined as meeting the three-variable 28-joint Disease Activity Score (DAS28) remission criterion at both the 3- and 12-month assessments [28], because of incomplete response, primary non-response, or secondary loss of efficacy [29,30,31]. Even when clinical remission or low disease activity is achieved, residual subclinical inflammation may persist, and disease flare remains common during tapering, discontinuation, or long-term follow-up [32,33]. In addition, although timely inflammatory control can reduce the radiographic progression, repair of established periarticular bone lesions is uncommon, and cartilage restoration remains limited, partly because the inflammatory microenvironment continues to impair tissue remodeling and repair [34]. These therapeutic shortcomings reflect, at least in part, the biological heterogeneity and signaling redundancy of RA, in which parallel inflammatory, stromal, and osteoclastogenic pathways can compensate when a single target is inhibited. Consequently, inhibition of selected mediators may attenuate disease activity, but often fails to provide comprehensive and durable control of the broader pathogenic network. This limitation is particularly relevant in RA disease, in which the effective and systematic intervention ideally requires not only suppression of overt inflammation, but also deeper modulation of immune–stromal crosstalk and protection against progressive structural damage.

The clinical utility of current RA therapies is further constrained by adverse effects and long-term safety issues, especially in the setting of chronic administration and combination treatment. Although these agents are indispensable for disease control, their benefits are often offset by cumulative toxicities and the need for ongoing safety monitoring. NSAIDs are associated with gastrointestinal, renal, and cardiovascular complications [35], whereas prolonged glucocorticoid use can lead to osteoporosis, metabolic disturbances, infection, and other systemic sequelae [36,37]. Conventional synthetic DMARDs may also cause hepatic, hematologic, pulmonary, or gastrointestinal toxicity, potentially limiting dose optimization or long-term persistence [25]. In addition, biologic agents and targeted synthetic therapies, despite their improved specificity, remain associated with infection risk and class-specific long-term safety concerns [38,39]. Thus, the long-term management of RA continues to involve a difficult balance between sustained efficacy and acceptable tolerability, highlighting the need for therapeutic approaches with broader applicability and improved safety profiles.

Beyond efficacy and safety, the current real-world effectiveness of RA therapy is also shaped by economic burden, unequal access to advanced agents, and challenges in long-term treatment adherence [40]. Biologic DMARDs and targeted synthetic therapies have substantially expanded treatment options, but their high costs limit timely initiation and sustained use [41,42]. In addition, access to these agents remains uneven across regions and clinical settings, owing to differences in reimbursement policies, healthcare infrastructure, and drug availability [43]. Long-term adherence may be further compromised by treatment burden, the need for repeated injections or infusions, and the need for continuous laboratory monitoring and follow-up [44]. These real-world barriers highlight that therapeutic success depends not only on pharmacologic efficacy, but also on accessibility, affordability, and sustained patient adherence.

In brief, the above limitations underscore the need for developing therapeutic strategies that are not only effective and safe, but also extend beyond single-pathway suppression and are capable of broader immunomodulation, sustained disease control, and protection against progressive tissue damage. In this context, growing attention has been directed toward natural products as potential RA interventions, owing to their structural diversity and pleiotropic bioactivities. Unlike single-target drugs, many natural compounds [45] can exert coordinated effects on inflammatory signaling, immune regulation, oxidative stress, and bone or cartilage metabolism, thereby offering a theoretical advantage as natural medicines in modulating the multifaceted pathogenic network of RA. Moreover, their long history of medicinal use and broad biological activities of natural products have further stimulated interest in their development as complementary or alternative therapeutic candidates.

## 3. Natural Products as RA Therapeutic Agents

The complexity of RA pathogenesis and the persistent limitations of current therapies have made natural products a compelling area of anti-RA research and drug development. Their value lies not only in broad pharmacological activity, but also in the capacity of many natural agents to modulate multiple targets of the RA pathogenic network. More fundamentally, research in this field has evolved from the empirical use of chemically complex natural preparations to the isolation and characterization of structurally defined bioactive constituents. Traditional complex products, including herbal medicines, natural extracts, and venom-based preparations, represent an experience-based therapeutic foundation and the potential for multicomponent synergy in treating RA. By contrast, the defined natural compounds mark a methodological shift toward compositional clarity, mechanistic tractability, and more controllable translational development. Accordingly, the following sections summarize the natural products in RA along this research continuum, from traditionally used complex preparations to defined bioactive compounds, as a framework for understanding both their therapeutic potential and their significance for modern anti-RA drug discovery.

### 3.1. Traditionally Used Complex Natural Products in RA Treatment

#### 3.1.1. Plant-Derived Traditional Complex Natural Products

Plant-derived traditional complex natural products constitute an important empirical resource for RA intervention [46], particularly in East Asian medical systems where herbal medicines and natural extracts have long been used to alleviate arthralgia, inflammation, and functional impairment (Table 1). Unlike structurally defined single compounds, these preparations are characterized by chemical complexity and may exert anti-RA effects through multicomponent synergy across multiple pathogenic processes. This feature is of particular relevance to RA, a disease driven by intertwined inflammatory, immune, stromal, and osteolytic pathways. At the same time, however, the compositional heterogeneity of plant-derived traditional natural products complicates quality control, standardization, pharmacodynamic material identification, and mechanistic interpretation. Accordingly, their value in RA research lies not only in their therapeutic potential, but also in their role as empirical and material foundations for the discovery of more precisely defined anti-RA bioactive constituents.

*Tripterygium wilfordii* Hook. f. (*Tw*HF) is one of the most representative plant-derived traditional complex natural products used for RA treatment. Rooted in long-standing use in Chinese medicine for rheumatic and autoimmune disorders, *Tw*HF has remained a major focus of modern anti-RA research, particularly in the form of various *Tw*HF preparations, whose activity has been linked to diterpenoids and other bioactive constituents. Clinical studies, including randomized trials and meta-analyses, suggest that these preparations may improve joint symptoms and disease activity in RA. In parallel, preclinical studies have shown that *Tw*HF and its related extracts can alleviate arthritis severity in collagen-induced arthritis models, with reductions in inflammatory responses and joint damage [76]. At the same time, *Tw*HF exemplifies the dual nature of traditional complex natural products: its therapeutic effects may arise from chemically complex, multitarget actions, whereas its translational use is substantially restricted by toxicity concerns, especially hepatotoxicity, reproductive toxicity, and other organ-specific adverse effects [77,78]. Thus, *Tw*HF not only highlights the therapeutic promise of plant-derived traditional complex natural products in RA, but also underscores the importance of rigorous standardization, safety evaluation, and pharmacodynamic material characterization in their further development.

*Sinomenium acutum* is a traditionally used anti-rheumatic medicinal plant in East Asian medicine and represents another typical plant-derived complex natural product for RA intervention [79]. Long employed for the treatment of rheumatic pain and inflammatory joint disorders, *S. acutum* has attracted sustained pharmacological interest, and its anti-RA activity has been attributed in part to sinomenine, the major alkaloid constituent of the plant [80]. Preclinical studies of sinomenine have identified anti-inflammatory and immunoregulatory effects, including suppression of nitric oxide production, inhibition of NF-κB signaling, and modulation of the Th17/Treg balance [81]. These findings characterize its experimental pharmacology but do not establish the mechanisms underlying any clinical benefit. In collagen-induced arthritis and related experimental models, sinomenine alleviates joint swelling, inflammatory responses, and joint injury [57,82], while clinical studies have also suggested benefits in improving joint symptoms and functional outcomes in RA [83]. From the perspective of natural-product research, *S. acutum* is particularly noteworthy because it illustrates the transition from empirical herbal use to modern pharmacological investigation: its traditional therapeutic value provides an important experiential basis, whereas the identification of sinomenine helps connect this complex medicinal plant to more clearly defined anti-RA bioactive compounds.

*Curcuma longa*, *Glycyrrhiza uralensis*, *Scutellaria baicalensis*, and *Paeonia lactiflora* are also frequently incorporated into traditional herbal formulations used for treatment of RA and other inflammatory arthropathies, reflecting their long-standing empirical relevance in anti-rheumatic therapy. *C. longa* has long been valued for its anti-inflammatory effects, and modern studies indicate that turmeric extracts and related preparations can alleviate pain and inflammatory severity in arthritis, with curcuminoids regarded as an important material basis for these effects [84]. *G. uralensis*, a classical medicinal plant rich in flavonoids and triterpenoid saponins, has also attracted attention in RA treatment research, as its preparations and representative constituents show anti-inflammatory and anti-angiogenic activities in experimental arthritis-related settings [65]. Similarly, *S. baicalensis* is widely recognized for its anti-inflammatory properties, and increasing evidence suggests that its flavone-rich extracts may suppress the pathogenic synovial responses and inflammatory signaling relevant to RA [85]. *P. lactiflora*, particularly in the form of total glucosides of peony, has been extensively used in East Asian medicine for inflammatory and immune disorders, and both experimental and clinical studies support its value in alleviating RA symptoms and modulating immune dysfunction [86,87].

Beyond these medicinal herbs, plant seed oils containing γ-linolenic acid (GLA), including evening primrose, borage seed, and blackcurrant seed oils, provide another clinically investigated category of complex botanical preparations. A Cochrane systematic review identified seven trials of GLA-containing oils and reported moderate evidence supporting symptomatic benefit in RA, including reduced pain and improved physical function [88]. Taken together, these herbal medicines further illustrate how traditionally used plant-derived complex natural products can serve as both empirical therapeutic resources and important material foundations for the subsequent identification of defined anti-RA bioactive compounds.

#### 3.1.2. Animal-Derived Traditional Complex Natural Products

Animal-derived traditional complex natural products represent a smaller but distinctive category of empirical bioresources for RA intervention. Compared with plant-derived herbal medicines, their diversity is more limited, but they provide complex natural components with characteristic anti-inflammatory, analgesic, and immunomodulatory properties. In the context of RA, the most representative examples are venom-derived preparations and lipid-rich natural extracts (Table 2), which may exert therapeutic effects through multiple bioactive components acting in concert. At the same time, however, their biological complexity also poses challenges for compositional standardization, mechanistic clarification, safety evaluation, and translational controllability.

Bee venom is the most intensively studied animal-derived complex natural product in RA treatment research. Traditionally used in East Asian medicine and in bee venom acupuncture, it is a chemically complex secretion enriched with bioactive peptides and enzymes, among which melittin and phospholipase A2 are regarded as major active components. Experimental studies suggest that bee venom and related preparations can attenuate arthritic inflammation by modulating key inflammatory mediators and signaling pathways, including NF-κB, TNF-α, IL-1β, and COX-2 [102,103,104]. Clinical studies and systematic reviews have also suggested that bee venom-based interventions may alleviate joint pain and swelling in RA, and safety concerns, particularly hypersensitivity reactions, require careful consideration [89]. Similar observations have been reported for snake venom-derived preparations, which have shown anti-inflammatory, analgesic, and immunomodulatory activities in experimental arthritis settings [97], but their application in RA remains far less established and is further constrained by toxicity and translational uncertainty. Thus, venom-derived natural products illustrate both the therapeutic promise and the developmental complexity of animal-derived traditional natural products in RA.

Fish oil represents another important animal-derived complex natural product with relevance to RA, particularly as an adjunctive nutritional intervention. As a lipid-rich marine-derived mixture, fish oil is characterized by high levels of omega-3 polyunsaturated fatty acids, especially eicosapentaenoic acid and docosahexaenoic acid, which contribute to its anti-inflammatory properties [105,106]. In RA-related studies, fish oil and omega-3 supplementation have been associated with modulation of macrophage function, suppression of pro-inflammatory lipid mediator synthesis, and reduction in synovial inflammatory burden [107]. Clinical evidence further suggests that omega-3-rich fish oil may improve pain, reduce tender and swollen joint counts, and lower the need for nonsteroidal anti-inflammatory drugs in some patients [98]. Compared with venom-derived preparations, fish oil is generally better suited to long-term adjunctive use; however, variability in formulation, dosage, and clinical response continues to limit its standardization and precise therapeutic positioning in RA management [108].

Animal-derived natural products have shown promising therapeutic potential in RA, primarily due to their potent anti-inflammatory and immunomodulatory effects. However, their clinical application faces significant challenges. One major limitation is immunogenicity, as these products can provoke immune responses that lead to adverse effects, especially with long-term use [109]. Additionally, many animal-derived compounds exhibit low oral bioavailability due to poor solubility and rapid degradation in the gastrointestinal tract, which limits their systemic efficacy [110]. The difficulty of large-scale extraction and the high cost of production further hinder their widespread clinical use [111].

Despite these challenges, recent biotechnological advances, such as peptide synthesis and recombinant expression systems, offer potential solutions. These technologies enable the production of bioactive compounds in a well-controlled, scalable manner, addressing some of the limitations of traditional extraction [112]. However, these methods are still in the developmental phase and have not yet reached broad clinical application.

#### 3.1.3. Fungal-Derived Traditional Complex Natural Products

Fungi, especially medicinal mushrooms and endophytic fungi, are increasingly recognized as valuable sources of natural anti-RA agents. These include bioactive polysaccharides, terpenoids, and secondary metabolites with immunomodulatory and anti-inflammatory activities.

*Ganoderma lucidum*, commonly known as reishi mushroom, is a widely studied medicinal fungus with well-established therapeutic effects. Its bioactive components, such as ganoderic acids and β-glucans, have been shown to exert potent anti-inflammatory, immunomodulatory, and antioxidant effects [113,114]. Studies indicate that extracts from *Ganoderma lucidum* can significantly reduce inflammation and pain in various arthritis models. For instance, in collagen-induced arthritis (CIA) models, *Ganoderma lucidum* extracts have been demonstrated to reduce joint swelling and prevent cartilage degradation by suppressing pro-inflammatory cytokines like TNF-α, IL-1β, and IL-6, and by modulating immune cell activity, including enhancing Treg responses and inhibiting Th17 cell activation [115]. Additionally, *Ganoderma lucidum* has been shown to regulate macrophage polarization, contributing to the reduction in oxidative stress and inflammation in synovial tissues [116]. These findings underscore the potential of *Ganoderma lucidum* as a therapeutic agent for RA, particularly in combination with other conventional therapies.

Similarly, *Cordyceps sinensis*, another medicinal fungus, has shown promising anti-inflammatory and immunomodulatory effects in RA therapy [117,118]. Its bioactive compound, *Cordyceps* polysaccharides, has demonstrated significant efficacy in reducing synovial inflammation and joint destruction in CIA models. The mechanisms of action include the suppression of NF-κB and MAPK signaling pathways, which are key regulators of inflammation and immune cell activation [119].

The diversity of fungal-derived natural products, including those from endophytes isolated from RA-related medicinal plants, represents a promising but underexploited therapeutic niche. Further studies are warranted to evaluate their pharmacokinetics, safety, and combinatory potential with plant- or drug-based therapies [120]. Compared to plant-derived compounds, fungal metabolites often have higher structural complexity and unique modes of action. However, the field remains nascent, with limited in vivo and clinical validation. Modern fungal genomics and metabolomics platforms may help unlock the therapeutic potential of this underutilized kingdom [121].

### 3.2. Defined Bioactive Natural Compounds in RA Therapy: Extraction, Structure and Mechanism

Natural products encompass a wide range of bioactive compounds with distinct chemical structures and pharmacological properties. These compounds can be categorized into several major classes—flavonoids, alkaloids, terpenoids, polysaccharides, saponins, and phenolic acids—each contributing uniquely to the mitigation of RA pathology [122]. Their mechanisms of action often include suppression of inflammatory mediators, oxidative stress inhibition, immune cell modulation, and cartilage protection (Table 3).

#### 3.2.1. Flavonoids

Flavonoids are a large class of polyphenolic compounds found in plants, known for their wide range of biological activities [164]. Structurally, they consist of a 15-carbon skeleton with two benzene rings (A and B) connected through a heterocyclic pyran ring (C), forming a C6-C3-C6 structure. The variation in the substitution of hydroxyl, methoxy, and glycosyl groups on the basic flavonoid structure leads to a diverse range of flavonoids with distinct properties. Flavonoids are typically categorized into several subclasses, including flavones, flavonols, flavanones, isoflavones, and anthocyanidins, with specific variations in their chemical structures influencing their biological effects. These compounds are generally water-soluble, but their bioavailability can be limited due to their low solubility and rapid metabolism in the liver [165,166,167].

The extraction of flavonoids typically involves methods such as solvent extraction using alcohol, methanol, or acetone [168]. More efficient methods, including UAE [169], MAE [170], and SFE [171,172], offer improved yield, shorter extraction times, and better preservation of thermolabile flavonoids [173,174]. Additionally, flavonoids can be purified using chromatographic techniques like high-performance liquid chromatography (HPLC) [175,176], ensuring higher purity levels necessary for research and clinical applications.

Flavonoids are renowned for their potent biological activities, particularly their anti-inflammatory, antioxidant, and immunomodulatory effects [177,178]. One of the key features of flavonoids is their ability to modulate various signaling pathways, including NF-κB, MAPK, and JAK/STAT, which are pivotal in inflammatory responses [179,180,181]. Flavonoids inhibit the expression of pro-inflammatory cytokines like TNF-α, IL-1β, and IL-6, thus reducing chronic inflammation in diseases such as RA [182].

Several flavonoids have been identified as potential therapeutic agents for RA, each with unique mechanisms of action. Quercetin, one of the most extensively studied flavonoids, has shown significant promise in RA treatment [183]. It reduces the activation of NF-κB and MAPK pathways [184], which are crucial in the production of inflammatory cytokines such as TNF-α and IL-6. Additionally, quercetin modulates immune responses by balancing the Th17/Treg ratio [185], a critical factor in autoimmune diseases. Preclinical studies in CIA models have demonstrated that quercetin reduces joint swelling, inhibits cartilage degradation, and improves disease scores [124,186].

Luteolin, another flavonoid, has been reported to inhibit the activation of the P2X4 pathway and reduce the production of pro-inflammatory cytokines [187]. In RA models, luteolin also alleviates synovial inflammation and joint destruction by modulating the immune response and protecting cartilage [188]. Similarly, kaempferol [130,189] and apigenin [134] exhibit anti-inflammatory and antioxidant effects, with kaempferol particularly known for its ability to inhibit osteoclastogenesis and protect bone integrity.

In summary, flavonoids offer a multi-target approach to RA treatment by modulating key inflammatory pathways, oxidative stress, and immune responses. The diversity of their mechanisms makes them valuable candidates for adjunctive therapies, either alone or in combination with csDMARDs, to provide more effective and sustainable management of RA.

#### 3.2.2. Alkaloids

Alkaloids represent a diverse class of nitrogen-containing secondary metabolites characterized by their heterocyclic structures and basic nitrogen atoms, which confer high affinity for nucleic acids, enzymes, and receptors [190]. These compounds are widely distributed in medicinal plants and have long been recognized for their strong immunosuppressive, anti-inflammatory, and analgesic properties [191,192,193,194]. In the context of RA, alkaloids modulate key signaling pathways [195] such as NF-κB, MAPK, and JAK/STAT, regulate T-cell and B-cell responses [196], and suppress inflammatory cytokine production [197].

The physical properties of alkaloids vary, with some being highly soluble in water, while others are fat-soluble and require lipid-based solvents for extraction [198,199]. Most alkaloids are alkaloidal salts, with a strong tendency to form complexes with acids. The extraction of alkaloids is typically carried out using organic solvents like ethanol, methanol, and acetone [200]. Modern extraction methods [201], such as SFE [202] and UAE [203], are also used to improve yield and reduce the time and cost of extraction. After extraction, purification can be achieved by techniques such as liquid chromatography, which helps isolate individual alkaloids for further study or clinical application [204].

In addition to sinomenine, other alkaloids also show promise. Matrine, a quinolizidine alkaloid isolated from Sophora flavescens, exerts anti-inflammatory effects by inhibiting the PI3K/AKT/mTOR pathway [139,205] and reducing osteoclast differentiation [206], thus preventing bone destruction in RA models [207]. Tetrandrine, a bis-benzylisoquinoline alkaloid from Stephania tetrandra, acts as a calcium channel blocker and reduces synovial fibroblast proliferation while downregulating inflammatory mediators [141,208,209]. Clinical observations indicate that tetrandrine can ameliorate RA symptoms when used as part of multi-herbal formulations [210].

The structural diversity of alkaloids provides multiple pharmacological entry points for RA intervention. However, their potency is often coupled with narrow therapeutic windows due to off-target toxicity. For example, while triptolide’s electrophilic moieties enhance immunosuppressive efficacy, they also account for organ toxicity [211]. Conversely, sinomenine and matrine exhibit milder but clinically safer effects, highlighting the balance between activity and tolerability. Modern approaches—including combinatorial alkaloid formulations, semi-synthetic derivatives, and nanoformulation-based delivery—are being explored to optimize efficacy and minimize systemic toxicity.

In summary, alkaloids represent a structurally diverse and pharmacologically potent category of natural compounds with established roles in RA management. Continued integration of structural chemistry, mechanistic immunology, and pharmaceutical innovation will be essential to fully realize the therapeutic potential of alkaloids in RA.

#### 3.2.3. Terpenoids

Terpenoids, also known as isoprenoids, constitute one of the most structurally diverse groups of natural products, synthesized from five-carbon isoprene units arranged in various configurations. The basic structure of terpenoids consists of a hydrocarbon backbone with varying levels of cyclization. Structurally, terpenoids are classified based on the number of isoprene units they contain, with the most common classes being monoterpenes (C10), sesquiterpenes (C15), diterpenes (C20), and triterpenes (C30) [212]. These compounds can be found in essential oils, resins, and various other plant and animal products [213].

Terpenoids’ physical properties, such as solubility, volatility, and stability, are influenced by their molecular structure [214]. While many terpenoids are volatile and can be easily extracted from plants using steam distillation, others are non-volatile and require organic solvents like ethanol or hexane for extraction [215].

Terpenoids are highly valuable in traditional medicine and pharmacology due to their broad spectrum of biological activities [213]. Their polycyclic frameworks, often modified by oxidation, glycosylation, or the presence of quinone moieties, confer significant pharmacological diversity, including anti-inflammatory, antioxidant, and antimicrobial effects [216,217]. Their diverse structures enable them to interact with various biological targets, making them potent modulators of multiple signaling pathways involved in disease pathogenesis.

In particular, terpenoids are known for their ability to modulate inflammatory pathways such as NF-κB, MAPK, and JAK/STAT, which are central to the inflammatory response in autoimmune diseases like RA [218]. Through this modulation, terpenoids reduce the production of pro-inflammatory cytokines such as TNF-α, IL-1β, and IL-6, thus helping to alleviate chronic inflammation. Additionally, their antioxidant activity, by scavenging reactive oxygen species (ROS), mitigates oxidative stress, a key factor in RA and other inflammatory disorders [219,220].

Several terpenoids have shown great promise in preclinical and clinical RA studies. For example, triptolide, derived from Tripterygium wilfordii (Thunder God Vine), is a potent diterpenoid that inhibits NF-κB and MAPK signaling, leading to a reduction in inflammatory cytokine production and T-cell activation [221]. Although triptolide has demonstrated efficacy in alleviating RA symptoms, its use is limited by its hepatotoxicity and reproductive toxicity. Boswellic acid, derived from Boswellia serrata (Indian frankincense), is another triterpenoid with significant anti-inflammatory effects [222]. It inhibits the 5-lipoxygenase enzyme, reducing leukotriene production and promoting cartilage protection, making it a potential adjunctive therapy for RA.

Moreover, ginsenosides, which are triterpenoid saponins found in Panax ginseng, exhibit anti-inflammatory and immunomodulatory effects by modulating several inflammatory pathways. They help reduce pro-inflammatory cytokine production and promote regulatory T cell differentiation, which is crucial for restoring immune balance in RA [154,223,224,225,226]. Similarly, andrographolide, a diterpenoid from Andrographis paniculata, has shown promise in inhibiting Th17 cell differentiation and suppressing NF-κB and MAPK signaling, offering another avenue for RA therapy [227,228].

Collectively, terpenoids represent a promising class of natural products for the treatment of RA due to their ability to modulate multiple inflammatory pathways, reduce oxidative stress, and regulate immune responses. Despite their potential, challenges related to bioavailability, toxicity, and standardization remain. Ongoing research into their pharmacokinetics, formulation improvements, and combination therapies will likely enhance the clinical efficacy of terpenoid-based treatments for RA.

#### 3.2.4. Polysaccharides

Polysaccharides are long-chain carbohydrates consisting of multiple sugar units linked by glycosidic bonds. These natural compounds vary in structure, size, and branching, and they are primarily classified into homopolysaccharides (composed of one type of sugar) and heteropolysaccharides (composed of different types of sugars) [229].

Polysaccharides are primarily extracted from plant, fungal, or algal sources through water extraction, where polysaccharides dissolve in hot water and are subsequently purified through methods like precipitation or filtration. Extraction techniques such as UAE use ultrasonic waves to enhance the extraction efficiency, while MAE accelerates the extraction process using microwave radiation, often resulting in higher yields of polysaccharides [230,231]. In addition, SFE, particularly when using CO_2_ as a solvent, has been applied to extract polysaccharides from various sources with high purity, making it suitable for clinical and research-grade extractions [232,233,234].

Due to their molecular complexity, polysaccharides possess a wide range of physicochemical properties, including water solubility and gel-forming abilities. Many polysaccharides are negatively charged, which enables them to interact with positively charged proteins and immune receptors, facilitating the modulation of immune functions. Their high molecular weight, branched structures, and unique glycosidic linkages allow them to interact with pattern recognition receptors (PRRs) such as Toll-like receptor 4 (TLR4), Dectin-1, and complement receptors [235]. These interactions modulate both innate and adaptive immune responses, helping maintain immune homeostasis. Unlike smaller phytochemicals that primarily target intracellular signaling pathways, polysaccharides exert their effects via extracellular receptor engagement, influencing systemic immune regulation [236]. The structural features of polysaccharides, such as β-(1→3)-glucan backbones, heteropolysaccharide branching, and uronic acid substitution, play a crucial role in their biological activity [235,237,238,239].

As a result, polysaccharides demonstrate a broad spectrum of immunomodulatory effects, including the modulation of cytokine release, macrophage polarization, and the activation of regulatory T cells (Tregs), making them promising candidates for therapeutic use in autoimmune diseases like rheumatoid arthritis (RA) [240,241,242]. Several studies have shown that polysaccharides derived from various sources, including *Ganoderma lucidum* (reishi mushroom) and *Astragalus membranaceus* (astragalus), can enhance the differentiation of regulatory T cells (Tregs) while suppressing pro-inflammatory Th17 cells [113,243,244,245]. In addition to their immune-modulating effects, polysaccharides from medicinal plants such as *Astragalus membranaceus* and Licorice root (*Glycyrrhiza uralensis*) also display significant antioxidant properties. By scavenging ROS, polysaccharides protect against oxidative stress, a major contributor to tissue damage and disease progression in RA [246]. For instance, *Astragalus polysaccharides* (APS) have been shown to improve antioxidant enzyme activity and reduce oxidative stress markers, thus alleviating joint swelling and cartilage destruction in animal models of RA [247]. Similarly, polysaccharides derived from *Fucoidan*, a sulfated polysaccharide from brown algae, exhibit potent antioxidant effects and have been found to reduce inflammation in RA models by suppressing ROS production and protecting against cartilage degradation [248,249,250].

## 4. Emerging RA Therapeutic Strategies Using Natural Products

Despite extensive preclinical evidence, the clinical translation of natural products in RA remains constrained by uncertain long-term efficacy and product-specific safety concerns. Randomized controlled trials and systematic reviews/meta-analyses report improvements in symptoms, disease activity, or medication requirements for some preparations (Table 4), but methodological limitations and heterogeneous treatment regimens restrict the certainty and generalizability of these findings. No natural product reviewed here has demonstrated inhibition of radiographic progression in an adequately powered, blinded trial.

Safety and compatibility with conventional treatment are equally important determinants of clinical utility. *Tw*HF preparations are associated with hepatotoxicity and reproductive toxicity, including menstrual irregularities, amenorrhoea, and reduced sperm counts; menstrual irregularities were also recorded in TRIFRA [251]. These effects make reproductive counselling and hepatic monitoring central to their clinical assessment. Bee-venom therapy carries a different but potentially life-threatening risk: severe hypersensitivity, including fatal anaphylaxis following live-bee acupuncture [252]. Herb–drug interactions also require compound-specific evaluation. Berberine inhibits CYP3A4 activity in humans [253], curcumin alters exposure to the P-glycoprotein substrate talinolol in human studies [254,255], and sinomenine modulates P-glycoprotein-mediated transport in an experimental model [256]. These findings warrant interaction testing with concomitant antirheumatic drugs, particularly CYP3A4-metabolized JAK inhibitors such as tofacitinib.

Product quality and regulatory status further determine whether trial findings can be translated into reproducible clinical practice. Batch-to-batch variation and risks of adulteration or contamination necessitate botanical authentication, chemical characterization, manufacturing controls, and testing for undeclared substances. Regulatory frameworks differ across jurisdictions: approved *Tw*HF preparations in China fall under national medicinal-product regulation; Japan regulates Kampo medicines through pharmaceutical approval and quality standards [257]; EMA herbal monographs inform national traditional-use registration or well-established-use authorization; and the FDA botanical drug pathway provides for investigational new drug applications and subsequent new drug applications.

**Table 4:** Clinical evidence from randomized controlled trials and systematic reviews/meta-analyses of natural products for rheumatoid arthritis.

Natural Product	Study Design	Intervention, Comparator and Duration	Main Outcomes	Ref.
*Tripterygium wilfordii* Hook F (*Tw*HF) extracts/preparations	Multicentre, double-blind RCT; n = 121	*Tw*HF extract (180 mg/day) versus sulfasalazine (2 g/day) for 24 weeks	ACR20 response: 65.0% versus 32.8% in the mixed-model analysis (*p* = 0.001). Radiographic progression was numerically lower with *Tw*HF, but the between-group difference was not significant.	[258]
	Multicentre, open-label RCT with blinded assessors (TRIFRA); n = 207	*Tw*HF (60 mg/day), MTX (12.5 mg/week), or their combination for 24 weeks; inadequate responders receiving monotherapy could switch to combination therapy at week 12	ACR50 response: 55.1% with *Tw*HF, 46.4% with MTX, and 76.8% with combination therapy; combination therapy was superior to MTX alone (*p* < 0.001).	[251]
	Two-year follow-up of TRIFRA; 109 of 207 participants completed follow-up; blinded radiographic assessment	Follow-up of the original *Tw*HF, MTX, and combination groups for 2 years	ACR50 at 2 years: 58.0% (*Tw*HF), 46.4% (MTX), and 50.7% (combination); *Tw*HF was non-inferior to MTX (*p* = 0.004).	[259]
	Multicentre, double-blind, triple-dummy RCT; n = 504	*Tw*HF + rhTNFR-Fc versus MTX + rhTNFR-Fc, *Tw*HF alone, or MTX alone; primary endpoint assessed at week 12	ACR50 response: 57.1%, 41.3%, 23.0%, and 26.2%, respectively. *Tw*HF + rhTNFR-Fc achieved higher response rates than each comparator (all *p* < 0.05).	[260]
	Systematic review and meta-analysis; 14 RCTs, n = 1446	*Tw*HF + MTX versus MTX alone; analyses included 12- and 24-week treatment courses	Combination therapy increased the reported effective rate (RR 1.15, 95% CI 1.10–1.21), partial remission rate (RR 1.27, 95% CI 1.15–1.40), and remission rate (RR 1.31, 95% CI 1.11–1.55).	[261]
	Three systematic reviews informing consensus guidance; 19 RCTs, n = 1795 overall; outcome-specific sample sizes	*Tw*HF alone versus MTX, and *Tw*HF + MTX versus MTX alone; treatment duration varied across trials	*Tw*HF alone showed no clear difference from MTX in ACR50 (3 RCTs, n = 419; RR 1.03, 95% CI 0.80–1.34; moderate certainty). Adding *Tw*HF to MTX improved ACR50 (4 RCTs, n = 500; RR 1.88, 95% CI 1.56–2.28; moderate certainty) and ACR70 (2 RCTs, n = 390; RR 2.12, 95% CI 1.40–3.19; low certainty).	[262]
*Sinomenium acutum*-derived sinomenine (SIN) preparations	Open-label, parallel-group RCT; n = 120; 101 completed 24 weeks	SIN (120 mg twice daily) + MTX versus leflunomide (20 mg/day) + MTX for 24 weeks	ACR50 response: 65.3% versus 69.6% in the intention-to-treat analysis, with no significant between-group difference. EULAR responses, CDAI, remission, and low disease activity rates also did not differ significantly.	[263]
	Multicentre, double-blind, double-dummy RCT; 135 enrolled; reported treatment groups comprised 38, 39, and 36 patients	Controlled-release SIN (120 mg twice daily), MTX (10 mg/week), or their combination for 24 weeks, with matching placebos	Primary endpoints were ACR50 response and CDAI improvement. 52.63% of SIN-treated patients achieved ACR50 at week 24; the authors reported comparable responses in the MTX and combination groups.	[264]
	Systematic review and meta-analysis; 16 RCTs, n = 1500	SIN alone versus MTX, or SIN + MTX versus MTX alone; treatment-duration range not specified in the abstract	The reported total effective rate favored SIN + MTX over MTX alone (*p* < 0.00001) and SIN alone over MTX (*p* = 0.04).	[265]
*Curcuma longa*-derived curcumin preparations	Randomized, double-blind, placebo-controlled, three-arm trial; n = 36	Bioavailable turmeric-matrix formulation (250 or 500 mg twice daily) versus placebo for 90 days	Mean DAS28 decreased from 4.51 to 2.14 with the lower dose and from 5.29 to 1.80 with the higher dose, while remaining 3.53 in the placebo group.	[266]
	Randomized, double-blind, placebo-controlled trial; n = 65 randomized	Curcumin nanomicelles (40 mg three times daily) versus placebo, added to existing medication, for 12 weeks	No significant between-group differences in changes in DAS28, tender joint count, swollen joint count, or ESR. Improvements within both groups did not establish an additional benefit of curcumin.	[267]
	Randomized, double-blind, placebo-controlled pilot trial; n = 30 analyzed	Nanomicellar curcumin (40 mg/day) versus placebo for 12 weeks	The between-group difference in DAS28-ESR improvement was not significant (*p* = 0.5125), although ESR and CRP reductions favored curcumin. Findings support an exploratory anti-inflammatory signal rather than established improvement in disease activity.	[268]
	Systematic review and meta-analysis of placebo-controlled RCTs; 6 studies, n = 244	Oral curcumin/curcuminoid formulations versus placebo; approximately 8–12 weeks; formulations and doses varied	The reported pooled DAS28 effect favored curcumin: SMD −3.40 (95% CI −5.29 to −1.50); 5 studies, n = 214. Heterogeneity was substantial (I^2^ = 96%), and GRADE certainty was very low.	[269]
*Paeonia lactiflora*-derived total glucosides of paeony (TGP)	Multicentre, open-label RCT; n = 268	TGP (1.8 g/day) + MTX (10 mg/week) + leflunomide (20 mg/day) versus the same MTX + leflunomide regimen without TGP for 12 weeks	Abnormal liver function occurred in 11.38% versus 23.26% (*p* = 0.013); ALT/AST > 3 times the upper limit of normal occurred in 1.63% versus 7.75% (*p* = 0.022). Remission and clinical response rates did not differ significantly.	[270]
	Systematic review and meta-analysis; 8 RCTs, n = 522	TGP (0.9–1.8 g/day) + MTX (7.5–15 mg/week) versus MTX alone for 12–24 weeks	Study-defined therapeutic response favored combination therapy: OR 3.70 (95% CI 1.51–9.04); 6 trials, n = 416. ESR was reduced by 5.85 mm/h (95% CI 3.02–8.67 lower).	[271]
	Systematic review and safety meta-analysis; 37 RCTs and 2 cohort studies, n = 3680 overall	TGP added to conventional treatment versus the corresponding regimen without TGP; background therapies and follow-up periods varied	Adjunctive TGP was associated with fewer hepatic adverse events (RR 0.31, 95% CI 0.23–0.41) and less leukopenia (RR 0.41, 95% CI 0.26–0.66). Gastrointestinal adverse events did not differ significantly (RR 1.05, 95% CI 0.90–1.23).	[73]
Bee venom (bee venom acupuncture and live bee-sting therapy)	Double-blind, placebo-controlled RCT; 80 randomized, 69 completed; subsequently evaluated in an RA-specific systematic review containing this single eligible RCT	Diluted bee venom injected into acupoints twice weekly for 8 weeks, versus normal saline injections at corresponding points	Improvements in pain and joint symptoms were reported. However, numerical estimates and significance statements were inconsistent across the review’s tables; a definitive effect size requires verification against the original trial. ACR/EULAR responses and DAS28 were not reported in the retrieved trial abstract. Safety reporting was insufficient. GRADE: low certainty in the systematic review, reflecting poor reporting and a small sample.	[89]
	Randomized controlled trial; n = 100, 50 per group; blinding not reported in the abstract	Live bee-sting therapy at selected points every other day, added to MTX, sulfasalazine and meloxicam, versus the same medication regimen alone; 3 months	Joint pain, swelling and morning stiffness favored adjunctive bee-sting therapy; reported relapse rates were 12% versus 32% (*p* < 0.05). Standardized ACR/EULAR responses, DAS28 effect sizes and confidence intervals were unavailable in the abstract. Adverse events were not adequately described.	[272]
	Randomized, active-controlled trial; n = 120, 60 per group; blinding not reported in the abstract	5–15 live bee stings per session, every other day, versus MTX 10 mg/week plus celecoxib 200 mg/day; 8 weeks	Study-defined response based on DAS28 was 83.33% versus 80.00% (*p* > 0.05); no significant between-group differences were reported for other clinical or inflammatory outcomes. No significant adverse effects were reported during treatment.	[273]
Marine fish oil (EPA/DHA-containing preparations)	Double-blind RCT in DMARD-naïve early RA; n = 140, high-dose group 87 and low-dose group 53	Fish oil providing EPA + DHA 5.5 g/day versus 0.4 g/day; both groups received algorithm-adjusted MTX + sulfasalazine + hydroxychloroquine; 12 months	High-dose fish oil reduced triple-DMARD failure: adjusted HR 0.24 (95% CI 0.10–0.54), and increased the rate of first ACR remission: adjusted HR 2.09 (1.02–4.30). DAS28, mHAQ and adverse events did not differ significantly.	[274]
	Two-center, double-blind, placebo-controlled RCT; 97 randomized, 58 completed	Cod liver oil 10 g/day, providing 2.2 g/day n-3 fatty acids, versus identical air-filled placebo capsules; 9 months; NSAID reduction was initiated after week 12	The primary endpoint, >30% reduction in daily NSAID requirement, was achieved by 19/49 (39%) versus 5/48 (10%); *p* = 0.002. Clinical disease activity and adverse effects did not differ significantly.	[275]
	Meta-analysis of 10 RCTs; n = 370	Omega-3 PUFA supplementation ≥2.7 g/day for ≥3 months, versus placebo; background treatment varied	NSAID consumption decreased: SMD −0.518 (95% CI −0.915 to −0.121; I^2^ = 0%). Effects on tender and swollen joint counts, morning stiffness and physical function were not statistically significant.	[276]
	Systematic review and meta-analysis of 23 placebo-controlled trials; total participant number not specified in the retrieved abstract	Oral n-3 fatty-acid supplements versus placebo; formulations, doses and durations varied across trials	Pooled effects were small and not statistically significant for pain: SMD −0.16 (95% CI −0.40 to 0.09); TJC: −0.20 (−0.46 to 0.05); and SJC: −0.10 (−0.28 to 0.07). NSAID reduction was also non-significant in sensitivity analysis. Evidence certainty was predominantly low or very low.	[277]

Advancing natural products for RA therefore requires an integrated approach combining rigorous clinical evaluation, systematic safety and interaction assessment, reproducible product quality, and a clearly defined regulatory pathway. Within this framework, multi-component combination therapies and advanced drug delivery systems address complementary therapeutic targets and compound-specific limitations in bioavailability, metabolic stability, and tissue exposure. Their translational value must be judged by demonstrable improvements in benefit–risk balance, rather than enhanced pharmacological activity alone.

### 4.1. Multi-Component Combination Therapies

RA treatment has significantly evolved, yet the management of this chronic autoimmune disease remains challenging. Traditional single-drug therapies, such as DMARDs and biologic agents, often face limitations related to their efficacy, side effects, and the development of immunogenicity. While DMARDs, including methotrexate, and biologics like TNF-α inhibitors have shown promising results, they are not universally effective, especially in patients with refractory disease. Additionally, the risk of severe side effects, such as hepatotoxicity and immunosuppression, further complicates long-term management [278]. These challenges underscore the need for alternative therapeutic strategies that can enhance the efficacy of current treatments while reducing their adverse effects.

In this context, natural product combination therapy has emerged as a promising approach. Natural products, with their diverse bioactive properties and multi-target mechanisms, offer a unique advantage in combination therapy. By targeting multiple pathways involved in the inflammatory and immune processes of RA, natural products can work synergistically with conventional drugs or even with other natural compounds to achieve better therapeutic outcomes. This approach helps overcome the limitations of single-drug therapy, including reducing side effects, enhancing efficacy, and minimizing the risk of drug resistance.

Combining natural products with conventional DMARDs is being investigated to improve disease control or mitigate treatment-related toxicity. Clinical support for this approach is provided by the TRIFRA trial. In this multicenter, open-label randomized study, TwHF plus methotrexate produced a higher ACR50 response rate at week 24 than methotrexate alone (76.8% versus 46.4%) [251]. Moreover, the combination of quercetin with MTX has been shown to alleviate methotrexate-induced hepatotoxicity while maintaining or even enhancing its anti-arthritic efficacy [279,280]. Quercetin’s antioxidant and hepatoprotective properties counteract MTX-related oxidative damage, allowing for prolonged therapy with fewer side effects. Similarly, curcumin administered with MTX or sulfasalazine has demonstrated synergistic suppression of inflammatory cytokines and reduced histological damage in preclinical models, while lowering the effective dose of the DMARDs [281]. Such strategies may help reduce long-term toxicity and improve patient adherence.

Dietary supplementation and nutraceuticals represent a rapidly expanding adjunctive strategy in the management of RA. The most common example of nutraceutical intervention is omega-3 polyunsaturated fatty acids (PUFAs), primarily eicosapentaenoic acid (EPA) and docosahexaenoic acid (DHA), derived from fish oil and marine sources. Omega-3 fatty acids act by displacing arachidonic acid in cell membranes, thereby reducing leukotriene and prostaglandin synthesis. They also promote M2 macrophage polarization and modulate T-cell activity [106,282]. Meta-analyses of heterogeneous randomized trials indicate small and inconsistent effects on pain and joint tenderness, with pooled estimates for several outcomes failing to reach statistical significance [277]. In an early-RA trial incorporating algorithm-based treat-to-target therapy, high-dose fish oil reduced triple-DMARD failure and increased ACR remission compared with low-dose fish oil, but did not improve DAS28 [274]. These outcome-specific findings do not establish a consistent disease-modifying effect. The 2022 ACR guideline on integrative interventions conditionally recommends following established dietary recommendations without adding dietary supplements for RA management [283], reflecting the absence of consistent, clinically meaningful benefits.

In conclusion, synergistic multi-compound and hybrid therapies offer an innovative paradigm for RA management and represent an exciting frontier, combining the best attributes of natural and synthetic agents. Future research should prioritize systematic evaluation of safety, pharmacokinetics, and clinical efficacy, alongside advances in formulation technologies, to realize the potential of synergistic strategies in RA therapy.

### 4.2. Advanced Drug Delivery Systems

The development of natural products for RA is constrained by compound-specific limitations in solubility, metabolic stability, and systemic exposure [284,285]. Advanced delivery systems are being investigated to improve drug exposure at inflamed joints while limiting systemic toxicity. Current approaches include nanocarriers, hydrogels, and microneedles, with supporting evidence derived predominantly from preclinical arthritis models.

Polymeric nanoparticles and liposomes have been explored for delivering curcumin, triptolide, and resveratrol [286]. In experimental arthritis, curcumin-loaded nanoparticles have improved drug exposure and attenuated inflammatory cytokine production and oxidative stress [287,288]. Hydrogels provide an alternative approach to local delivery and sustained release [289]. For example, a deep eutectic solvent–hydrogel formulation enhanced the skin permeation of herbal constituents and reduced joint inflammation and cartilage damage in rats with collagen-induced arthritis [290]. Microneedle-mediated delivery of bee venom has likewise alleviated arthritis manifestations in animal models [291,292]. These findings support further evaluation of formulation-specific benefits but do not establish clinical efficacy or safety.

Translation to RA care requires evidence that improved delivery produces meaningful benefits beyond those of the unformulated agent. Priorities include reproducible joint exposure, safety during repeated administration, manufacturing consistency, and compatibility with background DMARD therapy. Clinical studies must determine whether these systems improve disease control or tolerability, as favorable pharmacokinetics and efficacy in animal models alone are insufficient to establish therapeutic value.

## 5. Conclusions and Prospects

RA remains a challenging autoimmune disease characterized by chronic synovial inflammation, cartilage and bone destruction, and systemic comorbidities. Despite major advances in synthetic and biologic DMARDs, long-term management continues to be constrained by limited efficacy in refractory patients, toxicity, high costs, and the risk of opportunistic infections. Against this backdrop, natural products derived from plants, fungi, and animals have emerged as promising adjuncts or alternatives, offering broad-spectrum immunomodulatory, anti-inflammatory, antioxidant, and chondroprotective activities. As summarized in this review, diverse classes—including flavonoids, alkaloids, polysaccharides, terpenoids, saponins, and phenolic acids—exert their therapeutic effects by targeting multiple pathways such as NF-κB, JAK/STAT, MAPK, PI3K/AKT, and Nrf2, as well as modulating immune cell subsets including Tregs, Th17 cells, macrophages, and synoviocytes.

Compared to single-target synthetic drugs, natural products are distinguished by their multi-target pharmacology, which aligns well with the multifactorial pathogenesis of RA. Plant-derived compounds such as triptolide, celastrol, curcumin, quercetin, and resveratrol, as well as fungal polysaccharides from *Ganoderma lucidum* and *Cordyceps sinensis*, and animal-derived products such as bee venom and omega-3 fatty acids, have demonstrated efficacy in preclinical and, to varying extents, clinical settings. Moreover, novel strategies such as dietary supplementation, advanced delivery systems, and synergistic multi-compound therapies are expanding the therapeutic landscape. Together, these findings reinforce the translational promise of natural products in RA therapy.

Nevertheless, significant challenges impede their routine clinical application. First, pharmacokinetic limitations such as low oral bioavailability, poor solubility, and rapid systemic clearance are common among flavonoids, curcuminoids, and terpenoids. Second, toxicity concerns—as observed with triptolide and celastrol—pose barriers to long-term use, necessitating careful dose optimization and structural modifications. Third, quality control and standardization remain problematic, as the composition of herbal extracts can vary widely depending on species, cultivation conditions, and extraction methods. Fourth, while preclinical data are robust, high-quality randomized controlled trials (RCTs) remain scarce, and many clinical studies are limited by small sample sizes and heterogeneity in dosing regimens.

Looking ahead, several future directions hold promise for bridging the gap between bench and bedside:

1. Mechanistic insights through multi-omics and systems pharmacology: Integration of transcriptomics, proteomics, metabolomics, and network pharmacology enables the construction of comprehensive molecular interaction maps that delineate the complex relationships between natural products and RA-related pathways. This multi-omics approach not only enhances our understanding of the underlying mechanisms but also aids in the identification of potential biomarkers for predicting therapeutic response or treatment efficacy.
2. Innovative drug delivery systems: Nanoparticles, liposomes, micelles, hydrogels, and stimuli-responsive carriers have the potential to significantly enhance the solubility, stability, and targeted delivery of natural products. Particularly, intra-articular and localized delivery platforms are poised to not only improve therapeutic efficacy but also minimize systemic toxicity by offering controlled, site-specific release in response to the inflammatory microenvironment.
3. Synergistic and combination strategies: The rational design of multi-compound regimens, including both natural product–drug and natural product–natural product pairings, can harness additive or synergistic effects, potentially reducing adverse events by targeting multiple disease pathways. The development of hybrid molecules, which combine natural scaffolds with synthetic pharmacophores, represents an exciting frontier in medicinal chemistry, potentially yielding more potent and selective therapeutic agents.
4. Clinical translation and standardization: Large-scale, well-designed RCTs are needed to validate the therapeutic efficacy of natural products in RA, with a particular focus on optimizing dosing regimens and ensuring reproducibility across diverse patient populations. Additionally, regulatory frameworks must evolve to assess the safety, efficacy, and consistency of complex natural product formulations, ensuring their clinical translation while maintaining high standards for patient safety.
5. Precision nutrition and lifestyle integration: Given the growing recognition of the role diet and gut microbiota play in RA pathogenesis, nutraceuticals and functional foods, such as curcumin, omega-3 fatty acids, and polyphenols, can serve as preventive or adjunctive therapeutic strategies. Personalized nutrition, guided by pharmacogenomics and microbiome profiling, holds the potential to optimize therapeutic outcomes by tailoring interventions to an individual’s genetic makeup and microbial composition, thereby enhancing efficacy and minimizing adverse effects.
6. Integration of traditional knowledge with modern science and artificial intelligence (AI) advancements: Traditional medical systems, such as Traditional Chinese Medicine and Ayurveda, have long utilized natural products with therapeutic potential. Modern scientific methodologies, including clinical trials, molecular biology, and pharmacology, systematically validate these practices and uncover novel therapeutic leads. The integration of AI with multi-omics data (genomics, proteomics, transcriptomics) can identify new drug targets and therapeutic pathways that were previously difficult to discover. AI-driven algorithms, such as machine learning and deep learning, analyze biological data to predict interactions between natural products and disease mechanisms, revealing biomarkers for therapeutic response. AI also aids in optimizing drug formulations and delivery systems by simulating molecular interactions, enhancing treatment efficacy, particularly for RA. In precision medicine, AI can customize therapeutic regimens based on individual genetic, environmental, and lifestyle factors, ultimately improving treatment outcomes.

**Figure 2:**
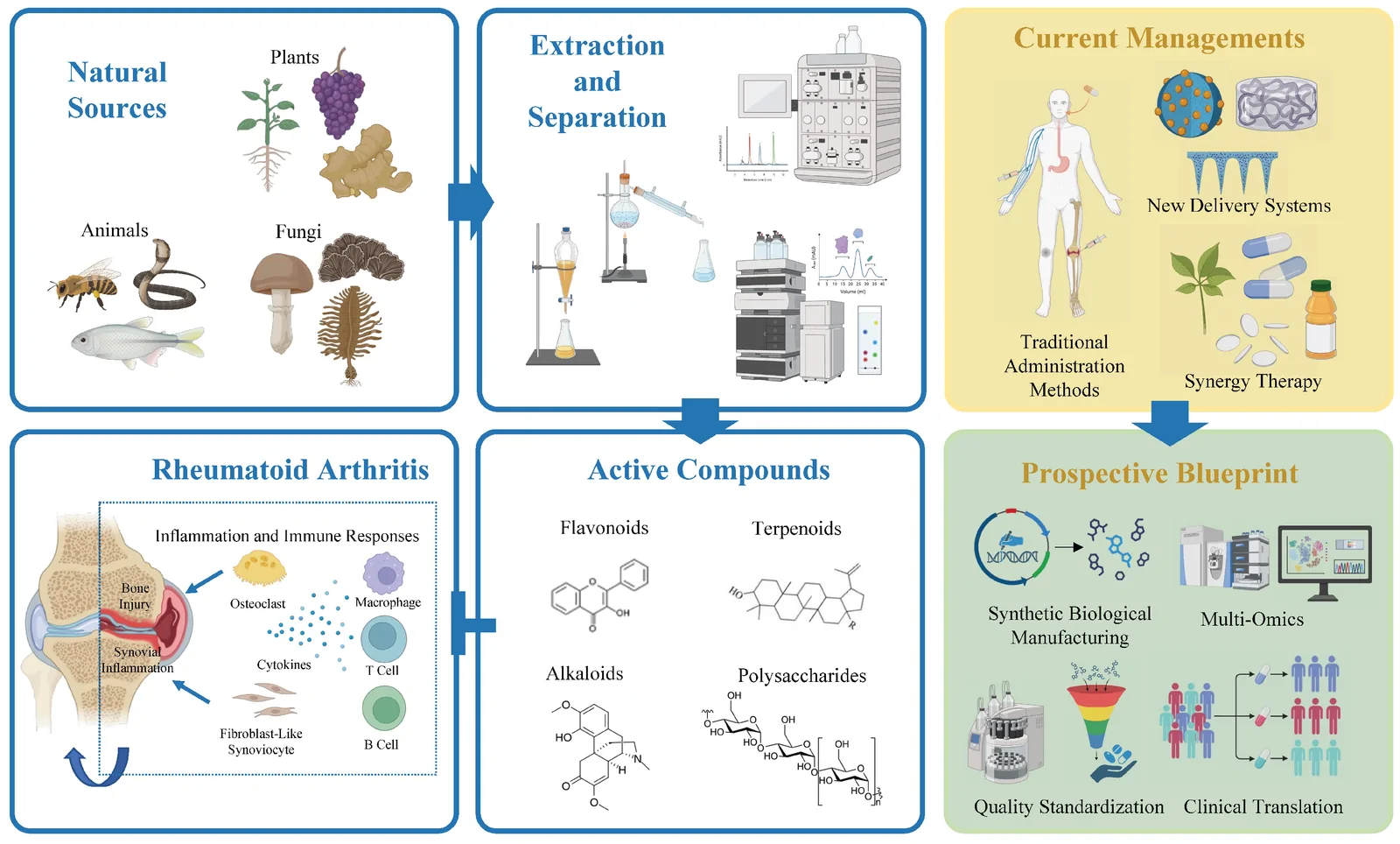
Overview of natural products in rheumatoid arthritis treatment and prospects. Plant-, animal-, and fungus-derived materials provide complex preparations and structurally defined constituents for investigating inflammatory, immune, and joint-destructive processes. Extraction, separation, and characterization establish the chemical basis for mechanistic studies, while combination therapies and advanced delivery systems seek to improve therapeutic performance. Further translation requires integration of synthetic biology, multi-omics, and quality standardization with rigorous clinical evaluation.

In conclusion, natural products hold immense promise as part of an integrated therapeutic paradigm for RA, complementing existing DMARDs and biologics. While challenges related to bioavailability, toxicity, and clinical validation remain, advances in chemistry, nanotechnology, and systems biology are rapidly creating new opportunities. By combining traditional knowledge with cutting-edge science, and by moving toward multi-target and precision-based approaches, natural product research may help to reshape RA management in the coming decades (Figure 2). The future of RA therapy may well lie in hybrid strategies that integrate synthetic drugs, biologics, and optimized natural compounds into a holistic, patient-centered framework.

## Figures and Tables

**Table 1:** Overview of plant-derived traditional complex natural products investigated for rheumatoid arthritis treatment.

Plant Name	Active Components	Research Models (Cell)	Research Models (Animal)	Effects	Region of Application	Ref.
*Tripterygium wilfordii* Hook. f. (Thunder God Vine)	Triptolide Celastrol	Human FLS Dendritic cells T-cells Synoviocytes Macrophages	CIA mice CIA rats	Suppresses NF-κB signaling; inhibits T-cell proliferation; reduces cytokine production Reduces ROS, IL-1β and TNF-α; alleviates paw swelling; protects cartilage	China, East Asia (Traditional Chinese Medicine)	[47,48,49,50,51,52,53]
*Sinomenium acutum* (Chinese Moonseed)	Sinomenine	RA-FLS Macrophages Neutrophils	CIA rats AIA mice	Inhibits MMP-9, TNF-α and IL-6; reduces angiogenesis and joint inflammation; improves joint function	China, Japan (Traditional Chinese Medicine, Kampo)	[54,55,56,57,58,59]
*Curcuma longa* (Turmeric)	Curcumin	RAW 264.7 macrophages RA-FLS Human RA PBMCs	CIA rats	Suppresses JAK/STAT, NF-κB, MAPK; downregulates TNF-α, IL-1β, IL-6 Reduces joint swelling and cartilage damage; prevents osteoclastogenesis	India, China, Global (Ayurveda, Nutraceutical)	[60,61,62,63,64]
*Glycyrrhiza uralensis* (Licorice)	Glycyrrhizin Liquiritin	Macrophages RA-FLS	CIA mice	Suppresses angiogenesis; inhibits MAPK signaling pathway; regulates gut–joint axis	China, Japan, Korea (Traditional Chinese Medicine, Kampo)	[65,66,67]
*Scutellaria baicalensis* (Baikal skullcap)	Baicalin Epiberberine	RA-FLS Macrophages Synoviocytes	CIA mice CIA rats	Anti-inflammatory, antioxidant; inhibits MAPK/NF-κB Reduces cartilage damage; antioxidant activity	China, Korea, Japan, and increasingly in Western countries (Traditional Chinese Medicine, complementary medicine)	[68,69,70,71,72]
*Paeonia lactiflora* (White peony)	Paeoniflorin	RA-FLS Osteoclasts	CIA mice	Reduces inflammatory cytokines; modulates immune balance	China, Japan, Korea (Traditional Chinese Medicine, Kampo)	[73,74,75]

**Table 2:** Overview of animal-derived traditional complex natural products investigated for rheumatoid arthritis treatment.

Source	Active Components	Research Models (Cell)	Research Models (Animal)	Effects	Region of Application	Ref.
Bee venom (Apis mellifera)	Melittin Apamin Phospholipase A2	Human synoviocytes Macrophages T-cells	CIA mice AIA rats	Inhibits NF-κB signaling; reduces TNF-α, IL-1β, COX-2 expression Alleviates joint pain and swelling in RA patients	China, Korea, Russia, and parts of Europe and South America	[89,90,91,92,93,94,95]
Snake venom (Viperidae, Elapidae)	Agkistrodotoxin Crotamine	Neuronal cells Rat FLS	CIA rats AA rats	Inhibits MMP-9; analgesic and immunosuppressive effects; inhibits immune hyperactivation Reduces pain behaviors in arthritis models	India (Ayurveda) and China	[96,97]
Marine fish oil (e.g., salmon, sardine)	Omega-3 PUFAs (EPA, DHA)	Macrophages Mouse FLS Endothelial cells	K/BxN mice CIA mice	Modulates macrophage polarization; suppresses prostaglandins and leukotrienes; reduces synovial inflammation; decreases morning stiffness and NSAID use in patients	Europe, North America, and Asia	[98,99,100,101]

**Table 3:** Bioactive natural compounds in RA therapy.

Compound Categories	Specific Compounds	Chemical Structure	Extraction Methods	Mechanisms of Action	Ref.
Flavonoids	Quercetin	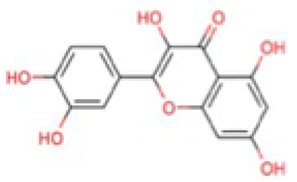 C_15_H_10_O_7_	Ultrasonic-assisted extraction, microwave-assisted extraction or Soxhlet extraction at optimized temperature around 60 °C	Inhibiting key signaling pathways like NF-κB, MAPK, and JAK1/STAT3/HIF-1α, which leads to decreased production of inflammatory cytokines (TNF-α, IL-1β, IL-6) and suppresses synovial fibroblast proliferation.	[123,124]
	Luteolin	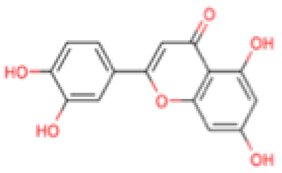 C_15_H_10_O_6_	Macerated extraction, solvent extraction, ultrasonic-assisted method, supercritical fluid extraction, microwave-assisted method	Inhibiting the NF-κB, MAPK, and Akt signaling pathways, reducing pro-inflammatory cytokines (IL-6, TNF-α, IL-1β), and inhibiting synovial fibroblast proliferation and matrix metalloproteinases (MMPs); suppresses neutrophil activation and Th17 cell differentiation.	[125,126,127]
	Kaempferol	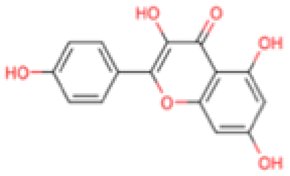 C_15_H_10_O_6_	Supercritical fluid extraction, molecular imprinting technique, ultrasound-assisted extraction, microwave-assisted extraction; optimal extraction often involves enzyme-assisted processes or SFE with CO at 25 MPa and 40 °C for high purity and yield	Induces apoptosis in activated synovial fibroblasts, suppresses inflammatory signaling pathways (PI3K/Akt, MAPK, NF-κB), and inhibits pyroptosis (NLRP3/CASP1/GSDMD axis).	[128,129,130]
	Apigenin	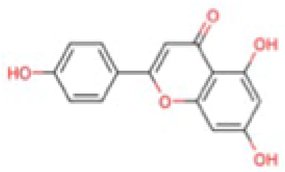 C_15_H_10_O_5_	Microwave-assisted or ultrasound-assisted extraction for high efficiency; optimal green methods use natural deep eutectic solvents, conventional methods utilize ethanol or ethyl acetate extraction followed by hydrolysis and purification	Inhibits NF-κB and MAPK pathways, reducing pro-inflammatory cytokines (IL-1β, IL-6, TNF-α), inhibits dendritic cell maturation, and regulates Th1/Th2 balance.	[131,132,133,134]
Alkaloids	Sinomenine	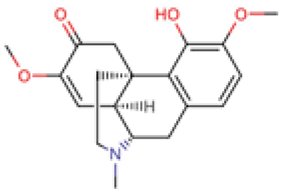 C_19_H_23_NO_4_	Ethanol reflux, acidic water extraction, and modern methods like deep eutectic solvents; optimal extraction techniques focus on achieving high purity (up to 96%) through techniques like centrifugal partition chromatography and ultrasound-assisted extraction	Inhibits pro-inflammatory cytokines (TNF-α, IL-6, IL-1β), suppresses synovial inflammation via NF-κB/MAPK pathway inhibition, reduces neutrophil migration/NETs formation, and protects against joint destruction by regulating osteoclast differentiation.	[79,135,136]
	Matrine	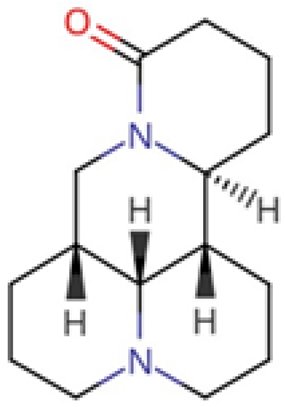 C_15_H_24_N_2_O	Solvent extraction, ultrasonic-aided extraction, and microwave-assisted extraction	Inhibits joint destruction by regulating inflammatory cytokine networks (IL-1β, TNF-α, IL-6), inhibits synovial angiogenesis (HIF-VEGF-Ang axis), reduces FLS proliferation, and induces apoptosis in RA-fibroblast-like synoviocytes.	[137,138,139]
	Tetrandrine	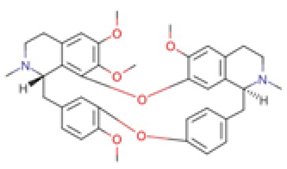 C_38_H_42_N_2_O_6_	Extracted from the root of Stephania tetrandra using alcohol-based, solvent-based, or ultrasound-assisted techniques, efficient extraction involves crushing the root, extracting with solvent (ethanol or supramolecular solvents), acidifying the extract, adjusting pH to ~9–11 for precipitation, and purifying with acetone or chromatography	Inhibits pro-inflammatory cytokines (IL-1, IL-6, IL-17), restricts FLS proliferation and migration, and reduces pannus formation.	[140,141,142]
Terpenoids	Boswellic acid	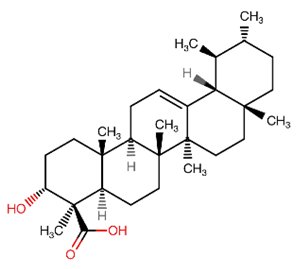 C_30_H_48_O_3_	Solvent-based methods, primarily ethanol or methanol, often followed by acid precipitation to enhance purity	Inhibits the 5-lipoxygenase (5-LOX) enzyme to reduce leukotriene synthesis, blocks the NF-κBpathway to reduce inflammatory cytokines, and inhibits matrix metalloproteinases (MMPs) that degrade joint cartilage.	[143,144,145]
	Triptolide	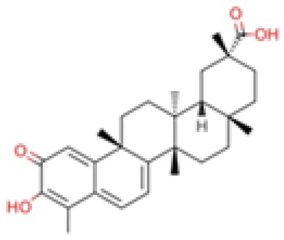 C_20_H_24_O_6_	Solvent extraction, usually with ethanol or ethyl acetate, followed by purification using silica gel column chromatography or High-Speed Counter-Current Chromatography (HSCCC)	Reduces synovial inflammation and joint destruction by inhibiting fibroblast-like synoviocytes (FLS) migration, inducing immune cell apoptosis (macrophages/dendritic cells), and regulating the Th17/Treg balance. Key signaling pathways targeted include NF-κB, MAPK, and JAK2/STAT3.	[146,147,148,149]
	Andrographolide	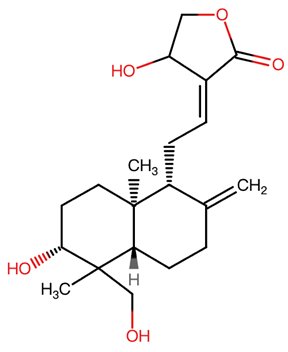 C_20_H_30_O_5_	Alcohol-based solvents, with methanol and ethanol generating the highest concentrations; optimal methods include using 80–95% ethanol for extraction, solvent-to-solid ratios of 10:1 or higher, and temperatures around 40–50 °C	Suppresses key signaling pathways (NF-κB, PI3K/AKT, MAPK), induces apoptosis in inflammatory synovial cells (RAFLSs), and inhibits neutrophil infiltration and NETosis.	[150,151,152]
	Ginsenoside	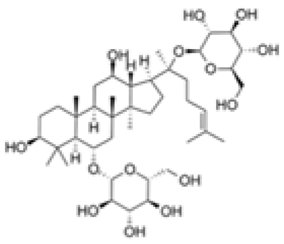 C_42_H_72_O_14_	Heat reflux extraction, ultrasound-assisted extraction, deep eutectic solvents, pressurized liquid extraction, ultrahigh pressure extraction	Inhibits the NF-κB and PI3K/Akt signaling pathway, reduces proinflammatory cytokines (TNF-α, IL-6), decreases synovial hyperplasia, and induces apoptosis in inflammatory cells.	[153,154,155,156]
Polysaccharides	Chitosan	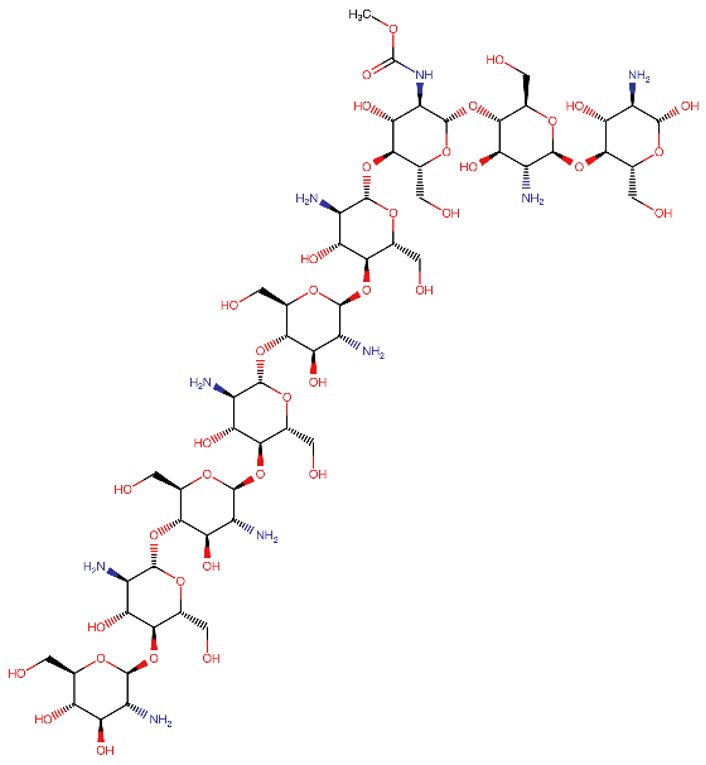 ${\left(C_{6}H_{11}O_{4}\right)}_{N}$	Demineralization (removing calcium carbonate with acid), deproteinization (removing proteins with alkali), and deacetylation (removing acetyl groups with strong alkali)	Acts as an anti-inflammatory, antioxidant, and immunomodulatory agent that reduces joint inflammation, suppresses pro-inflammatory cytokines (TNF-α, IL-6), and protects cartilage.	[157,158,159]
	Hyaluronic acid	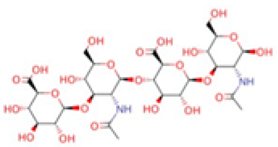 ${\left(C_{14}H_{21}NO_{11}\right)}_{N}$	Microbial fermentation (using Streptococcus bacteria) for high purity and yield, or extracted from animal tissues (rooster combs, bovine vitreous humor)	Reduces pain by improving synovial fluid viscosity, inhibiting inflammatory enzymes (like MMPs and COX-2), scavenging reactive oxygen species (ROS), and binding to CD44 receptors to suppress leukocyte migration.	[160,161,162,163]

## Data Availability

No new data were created or analyzed in this study. Data sharing is not applicable to this article.

## Abbreviations

The following abbreviations are used in this manuscript:

RA	Rheumatoid arthritis
DMARDs	Disease-modifying antirheumatic drugs
ACPA	Anti-citrullinated protein autoantibodies
MMPs	Matrix metalloproteinases
ADAMTS	A disintegrin and metalloproteinase with thrombospondin motifs
RANKL	Receptor activator of NF-κB ligand
NSAIDs	Nonsteroidal anti-inflammatory drugs
csDMARDs	conventional synthetic DMARDs
bDMARDs	biologic DMARDs
tsDMARDs	targeted synthetic DMARDs
JAK	Janus kinase
*Tw*HF	*Tripterygium wilfordii* Hook. f.
*C. longa*	*Curcuma longa*
*G. uralensis*	*Glycyrrhiza uralensis*
*S. baicalensis*	*Scutellaria baicalensis*
*P. lactiflora*	*Paeonia lactiflora*
CIA	Collagen-induced arthritis
APS	*Astragalus polysaccharides*
MTX	Methotrexate
PUFAs	Polyunsaturated fatty acids
EPA	Eicosapentaenoic acid
DHA	Docosahexaenoic acid
RCTs	Randomized controlled trials
DDS	Drug delivery systems
EPR	Enhanced permeability and retention
DES	Deep eutectic solvent
ACPA	Anti-citrullinated protein antibody
APC	Antigen-presenting cell
IFN-γ	Interferon-γ
IL	Interleukin
MHC II	Major histocompatibility complex class II
RANK	Receptor activator of nuclear factor-κB
RANKL	Receptor activator of nuclear factor-κB ligand
RF	Rheumatoid factor
TCR	T-cell receptor
AI	Artificial intelligence
